# Quantitative evaluation of breast density using a dual‐energy technique on a digital breast tomosynthesis system

**DOI:** 10.1002/acm2.12618

**Published:** 2019-05-20

**Authors:** Kun‐Mu Lu, Da‐Ming Yeh, Bi‐Hui Cao, Chia‐Yi Lin, Chih‐Yu Liang, Yu‐Bo Zhou, Chia‐Jung Tsai

**Affiliations:** ^1^ Department of Radiology Shin Kong Wu Ho‐Su Memorial Hospital Taipei City Taiwan; ^2^ Department of Medical Imaging Chung Shan Medical University Hospital Taichung City Taiwan; ^3^ Department of Medical Imaging and Radiological Sciences Chung Shan Medical University Taichung City Taiwan; ^4^ Department of Radiology The Second Affiliated Hospital of Guangzhou Medical University Guangzhou China; ^5^ Department of Information Engineering I‐Shou University Kaohsiung City Taiwan; ^6^ Department of Medical Imaging and Radiological Sciences I‐Shou University Kaohsiung City Taiwan; ^7^ Department of Emergency Medicine EDA Hospital, I‐Shou University Kaohsiung City Taiwan; ^8^ Department of Library Guangzhou Medical University Guangzhou China

**Keywords:** breast cancer, breast density, digital breast tomosynthesis, dual‐energy

## Abstract

**Purpose:**

Although breast density is considered a strong risk factor of breast cancer, its quantitative assessment is difficult. To investigate a quantitative method of measuring breast density using dual‐energy mammographic imaging with central digital breast tomosynthesis in physically uniform and nonuniform phantoms.

**Material and methods:**

The dual‐energy imaging unit used a tungsten anode and silver filter with 30 kVp for high‐energy images and 20 kVp for low‐energy images. Uniform glandular‐equivalent phantoms were used to calibrate a dual‐energy based decomposition algorithm. The first study used uniform breast phantoms which ranged in thicknesses from 20 to 70 mm, in 10‐mm increments, and which provided 30%, 50%, and 70% of breast density. The second study used uniform phantoms ranging from 10% to 90% of breast density. The third study used non‐uniform phantoms (at an average density of 50%) with a thickness which ranged from 20 to 90 mm, in 10‐mm increments.

**Results:**

The root mean square error of breast density measurements was 2.64–3.34% for the uniform, variable thickness phantoms, 4.17% for the uniform, variable density phantoms, and 4.49% for the nonuniform, variable thickness phantoms.

**Conclusion:**

The dual‐energy technique could be used to measure breast density with a margin of error of < 10% using digital breast tomosynthesis.

## 1 INTRODUCTION

Breast cancer is the most common cancer worldwide and is the most frequent cancer among women, with an estimated 1.67 million new cancer diagnoses in 2012 (25% of all cancers). It ranks as the fifth leading cause of death from cancer overall (5,22,000 deaths), as well as the most frequent cause of cancer death in women in less developed regions (3,24,000 deaths; 14.3% of total deaths). It is now the second leading cause of cancer death in more developed regions (1,98,000 deaths; 15.4% of total deaths), after lung cancer.^1^ Boyd et al hold the view that breast density, the percentage of glandular breast tissue, is a strong risk factor for breast cancer. A recent study has found that the risk of breast cancer is four to five times higher in women whose glandular density is higher than 75% than in those with breast density less than 25%.^2^ The possibility of predicting future disease occurrence in individuals could be applicable not only to the design and application of preventive plans but also to interventional trials and clinical decision making.^3^ Therefore, it is important to measure clinical breast density accurately and safely.

John Wolfe, a pioneer in the field of mammography, put forward a well‐cited theory of breast patterns as an index of risk for breast cancer in 1976, that of “breast patterns as an index of risk for developing breast cancer”.^4^ The current methods used to evaluate breast density include the four‐category Breast Imaging Reporting and Data System (BI‐RADS) classification system. BI‐RADS has demonstrated positive intrareader (k = 0.79–0.86) and interreader (k = 0.65–0.91) agreements, indicating interreader correlation (r) ranging from 0.7 to 0.93, with correlations better for D1 and D4 than for D2 and D3 breast density categories.^5^, ^6^ The reproducibility of BI‐RADS is generally poor owing to reader subjectivity in breast density assessment,^7^, ^8^ leading different implications regarding breast cancer risk prediction and choices in screening. To reduce variability and provide an objective measurement of breast density, quantitative approaches were developed for breast density evaluation.

Shepherd et al^9^ developed dual‐energy x‐ray absorptiometry (DXA) techniques to calculate density on both mammography units and bone densitometers.^10^, ^11^ Ducote et al subsequently investigated the feasibility of dual‐energy mammography to measure breast density in simulation and phantom studies.^12^, ^13^ Laidevant et al^14^ successfully regressed the protein composition and the thickness of lipid and water material in a phantom trial. These results can be applied directly to actual breast tissue to distinguish muscle, fat, and glandular components. However, the protocol for obtaining these values might not be applicable to clinical practice as it could possibly produce higher than recommended radiation doses that are not in compliance with international “as low as reasonably achievable” (ALARA) principles. The researchers mentioned above used uniform phantoms for their experiments; few researchers have focused on nonuniform materials and further studies are still necessary for heterogeneous bodies.

Digital breast tomosynthesis (DBT) is rapidly gaining in popularity worldwide due to its high spatial‐resolution tomographic images of the breast and its ability to create reconstructions using multiple low‐dose projection images.^15^ Early clinical trials show improved sensitivity and specificity of DBT compared with digital mammography (DM).^16^, ^17^ Bakic et al^18^ determined that breast density may be accurately estimated using central DBT and found substantial inter‐ and intrareader agreement in breast density estimation between DM and central DBT projection images. Our aim is to investigate the feasibility of the dual‐energy technique for quantifying breast density in uniform and nonuniform phantoms using a central DBT system.

## 2 MATERIALS AND METHODS

### 2.1 Dual energy decomposition and calibration

It is possible to combine low‐ and high‐energy images to enhance a particular component in a projection image. However, the presence of nonlinear effects precludes the use of linear log subtraction for generating accurate quantitative dual‐energy images. According to Kappadath et al,^19^ a linear function failed to model both the thickness and glandular ratio. On the contrary, quadratic, cubic, and conic functions could be adequately modeled. For this reason, a cubic model with 13 coefficients should be used for dual‐energy calibration. The cubic equation we used [eq. (1)] was:

(1) $$t=a_{1}+a_{2}A_{\mathrm{HE}}+a_{3}R+a_{4}T+a_{5}A_{\mathrm{HE}}^{2}+a_{6}R^{2}+a_{7}T^{2}+a_{8}A_{\mathrm{HE}}R+a_{9}A_{\mathrm{HE}}T+a_{10}RT+a_{11}A_{\mathrm{HE}}^{3}+a_{12}R^{3}+a_{13}T^{3}$$

where *t* (mm) represents the measured glandular thickness, *A*_HE_ the log‐signal functions for high‐energy, *R* the ratio of the log‐signal function for low‐energy and high‐energy (*R* = *A*_LE_/*A*_HE_), and *T* (mm) the total thickness, using a nonlinear least‐squares minimization algorithm (Levenberg‐Marquardt).^20^

Calibrations were carried out at clinically relevant breast thicknesses using pure adipose and pure glandular phantoms (Computerized Imaging Referencing Systems, Inc. [CIRS], Norfolk, VA, USA). Eighteen points were selected for dual‐energy calibration, which included uniform phantoms with thicknesses of 2–9 cm. The uniform phantoms represented either pure glandular tissue (100% density) or pure adipose tissue (0% density). These measurements were used to build a model and determine the coefficient index (Table 1) for eq. (1).

**Table 1:** Fitting coefficients of glandular tissue for dual‐energy calibration

Coefficient index	
a_1_	547.5679
a_2_	−3.9033
a_3_	−2227.0
a_4_	44.9791
a_5_	−3.0621
a_6_	2091.5
a_7_	−1.7670
a_8_	83.8225
a_9_	−5.0640
a_10_	−1.1086
a_11_	0.0671
a_12_	−792.6471
a_13_	0.0433

### 2.2 Phantom studies

Three phantom configurations of uniform‐ and nonuniform phantoms (model 014A and model 020, CIRS, Norfolk, VA, USA) were investigated. The first study used three groups of uniform phantoms (30%, 50%, and 70% density) with known thicknesses of 20–70 mm, in 10‐mm increments. The second study used uniform phantoms ranging from 10% to 90% density, at 5% intervals, with thickness varying from 15 to 100 mm (we used a 54% phantom rather than a 55% phantom due to material availability) (Table 2). The third study used nonuniform phantoms with an average density of 50%, ranging in thickness from 20 to 90 mm, in 10‐mm increments.

**Table 2:** Thicknesses and densities used in the second phantom study

Known density	Total thickness (mm)
10%	15
15%	20
20%	30
25%	20
30%	60
35%	20
40%	40
45%	60
50%	80
54%	25
60%	40
65%	100
70%	70
75%	60
80%	30
85%	20
90%	15

### 2.3 Image acquisition and processing

A commercial DBT unit (Selenia^TM^ Dimensions^TM^ System; Hologic, Bedford, MA, USA) was used for image acquisition. The device was equipped with a tungsten (W) anode x‐ray tube and x‐ray filters of rhodium (Rh), silver (Ag), and aluminum (Al). Different filters produce optimal x‐ray spectra on the basis of breast thickness, breast composition, and the desired imaging mode. In this study, an Ag filter was selected to increase spectral separation for the high‐energy beam in a dual‐energy composition.^12^ Central DBT imaging was acquired at 0‐degree projection with a spatial resolution of 70 μm per pixel using a detector with a 24 × 29‐cm^2^ field of view, corresponding to a 3328 × 4096 matrix. As in Feng and Sechopoulos,^21^ high‐energy images were set at 30 kVp and 100 mAs, a clinical protocol for an “average” breast. The low‐energy images were acquired at 20 kVp and 25 mAs, the lowest available setting on the DBT system.^22^ For central DBT, the breast phantoms were positioned for the craniocaudal view and were compressed using a standard force of 11 daN. We repeated this study three times during three months, acquiring a total of 129 measurements.

Mean glandular dose (MGD), the average value of absorbed dose in the breast with glandular tissue, was also used for an estimation of radiation‐induced breast cancer risk from mammography. It can be calculated from the eq. (2):

(2) $$\text{MGD}=\text{ESE}\times D_{\mathrm{gN}}$$

where ESE, i.e*.,* entrance skin exposure, is expressed in roentgens (R), and $D_{\mathrm{gN}}$ is the normalized dose conversion factor in mGy/R resulting from an incident exposure in air of 1R, being a function of breast density, breast thickness, X‐ray beam quality (i.e., tube potential and half‐value layer), and anode/filter combination.

All image processing was performed using MATLAB software, version 7.10.0.499 (MathWorks, Natick, MA, USA). All uniform and nonuniform materials were measured for both thickness and density, providing an accurate estimation.

### 2.4 Density measurement

As images were acquired and dual‐energy decomposition was performed, each pair of low‐ and high‐energy images was used to translate glandular and adipose material from pixels into thickness. A region of interest (ROI) was established for each image, and the mean glandular thickness (*T_g_*) was measured. The mean measured density (*D_m_*) was calculated by dividing the mean glandular thickness by the total thickness. This value was multiplied by 100 to convert the fractional density to a percentage [eq. (3)]:

(3) $$D_{m}\;\left(\%\right)=100\times \frac{T_{g}}{T}$$

**Figure 1:**
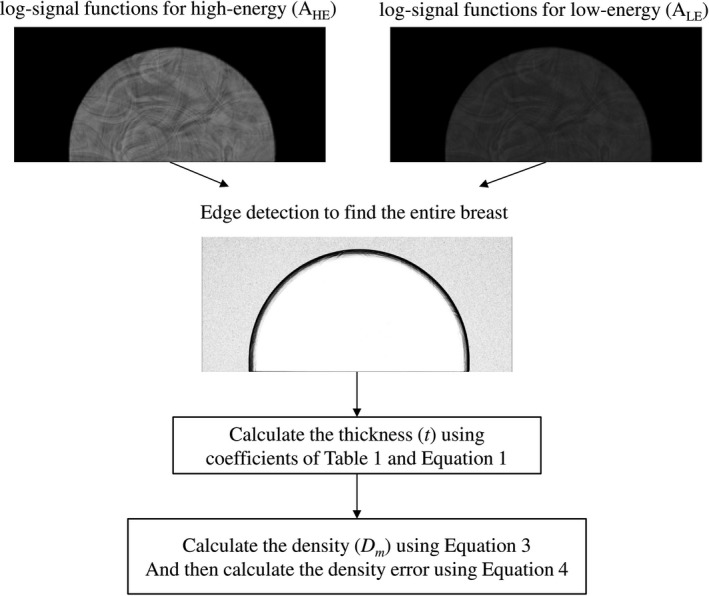
The flowchart of density measurements in this study

For the uniform‐thickness phantoms, the mean of each phantom image was sampled with a circular ROI (radius of 200 pixels) at the center‐of‐mass; the standard deviation (SD) was also measured. For the nonuniform phantom study, a user‐determined threshold (aimed at attempting to involve the entire phantom) was used when considering the heterogeneous features (Fig. 1). The process of selecting the ROIs was carried out on the high‐energy images, and this set of ROIs was used on both low‐ and high‐energy images without modification.

### 2.5 Error analysis

The root mean square error (RMSE) for density estimation was calculated using eq. (4):

(4) $$\text{RMSE}=\sqrt{\frac{\sum_{i=1}^{n}{\left(D_{m,i}-D_{k,i}\right)}^{2}}{n}}$$

where *D_k_* represented the known density.

## 3 RESULTS

**Figure 2:**
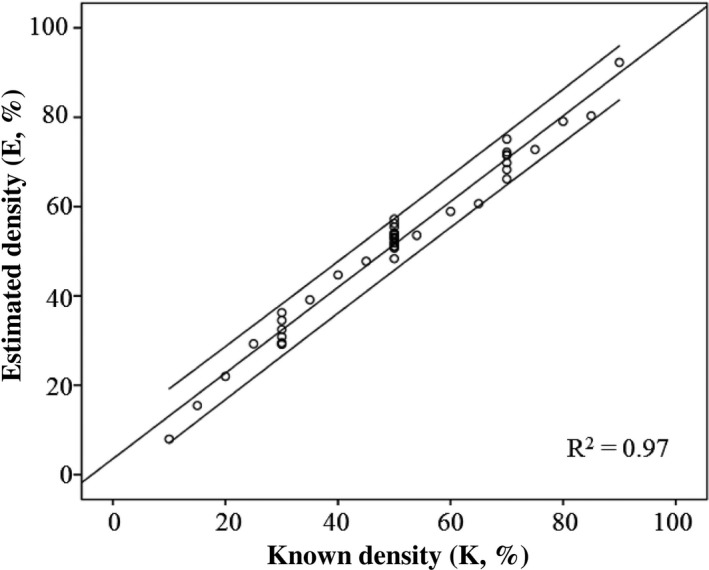
Density measurements for all three phantom studies. The known (K) and estimated (E) densities are related using E = 0.95 K + 3.54 with 95% confidence interval of 0.913–1.006. The goodness‐of‐fit (R^2^) value of linear regression was 0.97.

Results for the three phantom studies are tabulated in Tables 3, 4, 5. The measured glandular thickness and density are shown as the mean value ± SD; the calculating errors are represented by the RMSE. The thickness errors are displayed as the mean value (mm). For the first study (Table 3), the RMSE was 3.34% for 30% density, 2.64% for 50% density, and 2.89% for 70% density, while the corresponding mean measured density was 32.15%, 52.36%, and 71.06%, respectively. For the second study (Table 4), the RMSE was 4.17% for all known‐density phantoms. The glandular thickness and density errors were approximately 0.52 mm and 0.18%, respectively. For the third study, the results from the nonuniform phantoms (Table 5) had a mildly higher error value, with an RMSE of 4.49%; the averaged errors of measured glandular thickness were 2.70 mm. The averaged density error for all three studies was 3.66%. The results of the density measurements for all three studies are shown in the Fig. 2. The slope and goodness‐of‐fit (R^2^) values of linear regression were 0.95 (95% confidence interval, 0.913–1.006) and 0.97, respectively. The relation between known (K) and estimated (E) densities was calculated using the equation: E = 0.95 K + 3.54.

**Table 3:** Errors in density estimation, first study (uniform phantoms of 30%, 50%, and 70% density; root mean square error, 3.34%, 2.64%, and 2.89%, respectively)

Known density	Known thickness (mm)	Measured glandular thickness (mm)	Thickness error (mm)	Measured density (%)
30%	20	7.59 ± 0.56	1.59	34.54 ± 2.55
	30	11.23 ± 0.58	2.23	36.24 ± 1.87
	40	13.32 ± 0.82	1.32	32.48 ± 2.01
	50	15.05 ± 1.03	0.05	29.51 ± 2.03
	60	17.86 ± 1.32	0.14	29.28 ± 2.17
	70	21.90 ± 1.70	0.90	30.85 ± 2.40
50%	20	11.15 ± 0.41	1.15	50.69 ± 1.89
	30	16.92 ± 0.64	1.92	52.88 ± 2.01
	40	22.08 ± 0.75	2.08	53.85 ± 1.83
	50	26.98 ± 0.94	1.98	51.88 ± 1.82
	60	32.73 ± 1.12	2.73	53.65 ± 1.84
	70	36.34 ± 1.47	1.34	51.18 ± 2.07
70%	20	15.77 ± 0.27	1.77	75.11 ± 1.32
	30	21.17 ± 0.61	0.17	66.17 ± 1.92
	40	29.59 ± 0.70	1.59	72.18 ± 1.72
	50	36.54 ± 0.71	1.54	71.64 ± 1.41
	60	43.58 ± 0.91	1.58	71.44 ± 1.50
	70	50.26 ± 1.17	1.26	69.80 ± 1.49

**Table 4:** Errors in density estimation, second study (uniform phantoms; root mean square error, 4.17%)

Known density	Known glandular thickness (mm)	Measured glandular thickness (mm)	Thickness error (mm)	Measured density (%)	Density error (%)
10%	1.5	1.35 ± 0.11	0.15	7.93 ± 0.64	2.07
15%	3	3.40 ± 0.31	0.40	15.45 ± 1.40	0.45
20%	6	6.81 ± 0.60	0.81	21.99 ± 1.95	1.99
25%	5	6.44 ± 0.53	1.44	29.27 ± 2.40	4.27
30%	18	17.79 ± 1.32	0.21	29.17 ± 2.16	0.83
35%	7	8.61 ± 0.47	1.61	39.13 ± 2.13	4.13
40%	16	18.32 ± 0.81	2.32	44.69 ± 1.98	4.69
45%	27	29.12 ± 1.18	2.12	47.74 ± 1.94	2.74
50%	40	41.24 ± 1.76	1.24	50.92 ± 2.17	0.92
54%	13.5	14.46 ± 0.44	0.96	53.57 ± 1.65	0.43
60%	24	24.14 ± 0.71	0.14	58.90 ± 1.74	1.10
65%	65	61.27 ± 0.93	3.73	60.66 ± 0.92	4.34
70%	49	49.16 ± 1.17	0.16	68.28 ± 1.30	1.72
75%	45	45.12 ± 0.90	0.12	72.78 ± 1.45	2.22
80%	24	24.51 ± 0.56	0.51	79.08 ± 1.81	0.92
85%	17	16.86 ± 0.20	0.14	80.32 ± 0.97	4.68
90%	13.5	14.76 ± 0.48	1.26	92.28 ± 3.03	2.28

**Table 5:** Errors in density estimation, third study (nonuniform phantoms of 50% density; root mean square error, 4.49%)

Known thickness (mm)	Measured glandular thickness (mm)	Thickness error (mm)	Measured density (%)
20	12.37 ± 1.80	2.37	56.24 ± 8.18
30	16.60 ± 2.86	1.60	51.90 ± 8.96
40	23.45 ± 3.34	3.45	57.20 ± 8.15
50	27.52 ± 4.24	2.52	52.93 ± 8.16
60	32.94 ± 4.81	2.94	53.14 ± 7.76
70	39.89 ± 5.49	4.89	55.41 ± 7.62
80	44.38 ± 5.96	4.38	54.12 ± 7.25
90	44.47 ± 5.74	0.53	48.34 ± 6.24

## 4 DISCUSSION

Breast density is one of the strongest predictors of breast cancer risk. The extent of breast density can be modified by several factors. Increasing age and menopause^23^ are independent contributors to a decrease in breast density.^24^ Elevated body mass index has been associated with low breast density, whereas increased age at first childbirth has been associated with high breast density.^25^ Pregnancy at an early age decreases breast density, and this beneficial effect appears to be permanent.^26^ Postmenopausal hormonal therapies that include both estrogen and progesterone are associated with an increase in breast density that decreases upon discontinuation of therapy.^27^ Intervention trials have shown that decreases in breast density are associated with tamoxifen treatment.^28^ a therapy proven to decrease breast cancer risk. Therefore, some qualitative and quantitative methods involve grading of mammographic density.

Digital breast tomosynthesis is gaining approval as a tool for the evaluation of breast density as well as an adjunct to digital mammography in screening. Ekpo et al^29^ point out that the investigation and measurement of breast density has been evolving for more than 30 years. FFDM has been frequently used for earlier breast density surveys in simulation^12^ or phantom^13^ studies. However, comparing DBT to FFDM reveals several merits to the former, even though the latter is more commonly used. First, DBT shows a stronger correlation of breast density to the parenchymal texture features than DM.^30^ These results may be reasonable, as DBT takes the whole breast into consideration and acquires details of depth from the tomographic breast. Additionally, the recall rates for noncancer cases significantly decrease with the addition of tomosynthesis to DM.^31^, ^32^, ^33^ Our selection of DBT may be well founded, as our results are persuasive when combined with previous research. Ducote et al^13^ accurately used uniform phantoms to quantify breast density with a dual‐energy based decomposition algorithm. The present study also showed good functionality for the use of the dual‐energy technique for breast density evaluation in uniform and nonuniform phantoms using a central DBT system. Previous study by Ducote found a RMSE for breast density measurements of 0.44% (our range, 2.64%–3.34% for three glandular densities) for variable thickness phantoms and 0.64% (our value, 4.17%) for variable density phantoms. Although our results differed slightly, this might be explained by the two following reasons: (a) our study aimed to use a clinically feasible protocol; hence, our use of both high‐energy and low‐energy images (30 and 20 kVp [the lowest available on the Hologic DBT]) apparently reduced the separation in mean energy between the two beams. Ducote et al used 49 and 28 kVp for their images,^13^ resulting in lower errors. (b) A correction for x‐ray scatter was absent in our study, even though this is the predominant source of error in breast density measurement.^34^, ^35^ Encouragingly, even the nonuniform phantoms (well‐matched to actual breast tissue in density composition), in which we expected more systematic error, yielded a relatively ideal result with a RMSE value of only 4.49% (compared with 4.17% in uniform phantoms), even though this was the first used of a commercial DBT unit for breast density evaluation (Table 5).

**Figure 3:**
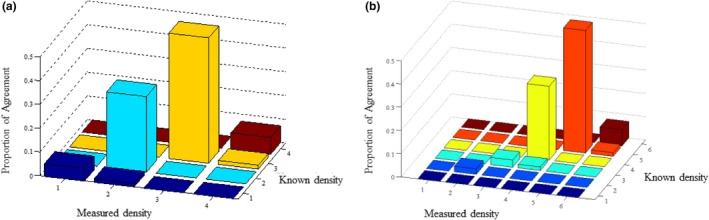
Graph shows consistent agreement between the categoric estimates of breast density using the Breast Imaging Reporting and Data System (BI‐RADS) classification (a) and the Boyd six‐grade classification (b)

At present, the classical Wolfe four‐grade (BI‐RADS) system and the Boyd six‐grade classification are indispensable in clinical breast density grading. The distribution of calculated breast density on central DBT with dual energy projections in the Wolfe and Boyd systems is shown in the Fig. 3. BI‐RADS and Boyd six‐grade classifications would result in four overestimations using our data. From the standpoint of BI‐RADS [Fig. 3(a)] and Boyd six‐grade [Fig. 3(b)] classification, the known glandular densities of 20% and 70% belong to class 1 vs class 3, and class 3 vs class 5, respectively, whereas the corresponding measured 25% and 76% densities in these categories can be assigned to class 2 vs class 4, and class 4 vs class 6. The overestimation in the lower and upper quadrants might result from the absence of scatter correction and the inherent higher recall rate for patients with dense breast tissue because of radiologic tumor masking.^2^ A previous study^18^ investigates this variance by measuring the percent density (PD) in 39 women using FFDM and DBT. Three readers estimated quantitative PD values and six‐class Boyd categories, and repeated the study after 8 months, showing a high correlation and substantial agreement between PD on DM and on central DBT projections. However, this study analyzed breast density with only the semiquantitative technique, and uncertainty in the correlation between DM and DBT should be considered due to subjective bias. In our study, the automatic quantitative method was employed, thus eliminating the subjective effect.

For 50% glandular density and a thickness of 5 cm, the total summarized MGD in dual‐energy images is 1.78 mGy for uniform and 1.81 mGy for nonuniform phantoms. By comparison, the average MGD for screening mammography in the United States in 2006 was 1.8 mGy for a reference phantom equivalent to 50% density and 4.7 cm thickness.^36^ In a study of five DM systems, the MGD varied from 1.4 to 2.4 mGy for 1‐view screening mammography.^37^ The dual‐energy DBT used in our study results in a dose comparable to that of routine DM. There are some limitations in this study; the first, regression coefficients in Table 1 such as *A*_HE_ or *R* value might be changed depending on detector type (Y6‐ or Y8), software version and calibration. So, other coefficients could be taken into consideration for modification breast density accuracy in the future. Another limitation is the absence of an x‐ray scatter correction, which is the predominant source of error in breast density measurement using dual energy mammography. In addition, we used a newly proposed algorithm for which more study is needed to verify efficacy. Future clinical implementation of this technique is expected using a clinical protocol in which automatic exposure control, as high‐energy imaging, is combined with low‐dose 3D projection, as low‐energy imaging. Previously, some 2D interactive computer programs have also been used to generate a percentage mammographic density.^5^, ^38^, ^39^ These methods, as well as other similar interactive computer and qualitative estimates, assess a 3D organ using 2D techniques, so are likely to be limited. Therefore, DBT potentially has sufficient superiority, which provides 2D and 3D imaging for diagnosis while offering quantification of breast density as a risk factor for breast cancer.

## 5 CONCLUSIONS

In conclusion, the RMSE for breast density measurements was 2.64–3.34% for uniform, variable thickness phantoms, 4.17% for uniform, variable density phantoms, and 4.49% for nonuniform, variable thickness phantoms. The results of these phantom studies indicate that the dual‐energy technique can be used to measure breast density with an error of < 10%, using DBT.

## CONFLICT OF INTEREST

The authors declare no competing financial interests.
